# A Novel Framework for Enhancing Decision-Making in Autonomous Cyber Defense Through Graph Embedding

**DOI:** 10.3390/e27060622

**Published:** 2025-06-11

**Authors:** Zhen Wang, Yongjie Wang, Xinli Xiong, Qiankun Ren, Jun Huang

**Affiliations:** 1 College of Electronic Engineering, National University of Defense Technology, Hefei 230037, China; wangzhen.123@nudt.edu.cn (Z.W.); xiongxinli_@nudt.edu.cn (X.X.); renqiankun@nudt.edu.cn (Q.R.); huangjun0111@nudt.edu.cn (J.H.); 2 Anhui Province Key Laboratory of Cyberspace Security Situation Awareness and Evaluation, Hefei 230037, China

**Keywords:** autonomous cyber defense, intelligent decision-making, graph embedding, reinforcement learning

## Abstract

Faced with challenges posed by sophisticated cyber attacks and dynamic characteristics of cyberspace, the autonomous cyber defense (ACD) technology has shown its effectiveness. However, traditional decision-making methods for ACD are unable to effectively characterize the network topology and internode dependencies, which makes it difficult for defenders to identify key nodes and critical attack paths. Therefore, this paper proposes an enhanced decision-making method combining graph embedding with reinforcement learning algorithms. By constructing a game model for cyber confrontations, this paper models important elements of the network topology for decision-making, which guide the defender to dynamically optimize its strategy based on topology awareness. We improve the reinforcement learning with the Node2vec algorithm to characterize information for the defender from the network. And, node attributes and network structural features are embedded into low-dimensional vectors instead of using traditional one-hot encoding, which can address the perceptual bottleneck in high-dimensional sparse environments. Meanwhile, the algorithm training environment Cyberwheel is extended by adding new fine-grained defense mechanisms to enhance the utility and portability of ACD. In experiments, our decision-making method based on graph embedding is compared and analyzed with traditional perception methods. The results show and verify the superior performance of our approach in the strategy selection of defensive decision-making. Also, diverse parameters of the graph representation model Node2vec are analyzed and compared to find the impact on the enhancement of the embedding effectiveness for the decision-making of ACD.

## 1. Introduction

In recent years, cyber attacks and defense have demonstrated a pronounced trend toward intelligent upgrades. On the attack side, attack methodologies have advanced from rudimentary intrusions by early script kiddies to sophisticated, intelligent agent-based attacks characterized by autonomous decision-making capabilities. Adversaries now employ deep reinforcement learning techniques to develop adaptive attack models, enabling them to intelligently select targets, dynamically allocate attack payloads, and autonomously plan attack paths. Consequently, this has exponentially increased the stealth and destructiveness of attacks.

Currently, cyber security defense technologies have evolved from static rule matching to dynamic, proactive defenses. Traditional defense strategies, which rely on manually defined static rules, such as those used in firewalls and intrusion detection systems that primarily depend on static signatures and patterns, struggle to adapt to dynamically evolving attack modalities. Using mechanisms such as Moving Target Defense (MTD) [1], Cyber Resilience (CyR) [2], and Proactive Cyber Deception (PCyD) [3], defenders actively and continuously modify the security factors and attributes of the system to prevent attacker activities. Despite these advancements, existing defense systems still face significant challenges in countering intelligent attacks. Inadequate representation of network topology and node attributes during the decision-making process hampers an agent’s ability to capture critical features of complex environments, thereby undermining its decision-making capability. Additionally, due to constraints such as network bandwidth and real-world network conditions, training autonomous cyber defense agents is typically conducted in simulated environments. The granularity of these simulation environments significantly limits the generalizability of defense models, and effectively transferring the action selection learned in simulations to real-world settings remains a critical challenge in cyber defense technology. In summary, autonomous cyber defense against intelligent attacks faces the following challenges:

- The network topology information significantly impacts the decision-making of intelligent agents. Traditional one-hot encoding is insufficient to represent network states, necessitating the construction of more granular observation vectors to support defender decision-making.
- Existing intelligent agent training and validation environments mostly support abstract simulations of cyberspace, lacking fine-grained simulations of real environments to enhance training authenticity and efficiency. It is essential to train our intelligent agents in high-fidelity simulated environments to ensure their transferability to real network topologies.

To address these challenges, this paper proposes an autonomous cyber defense decision method based on graph embedding, modeling attackers and defenders in a network as a game. The core idea is that the defense agent should choose the optimal defense strategy in a game between the attacker and the defender, continuously updating the strategy by observing the environment. A series of experiments were conducted to validate the effectiveness and efficiency of the proposed method. The main contributions are as follows:

- This paper constructed a game model based on the continuity, randomness, and dynamics of a network attacker and defender, quantifying both parties’ payoffs and strategy choices to provide theoretical support for autonomous defense decision-making.
- This paper improves the traditional one-hot coding method using graph embedding to map the node configuration information and network topology into a low-dimensional vector space for a better representation, greatly improving the decision-making capability of autonomous defense agents.
- We extended the action space of the blue team in the high-fidelity simulation environment Cyberwheel to allow it to perform more granular defensive actions that better align with real-world cyber defense scenarios.

The remainder of this paper is organized as follows. Section 2 reviews related work on autonomous cyber defense and reinforcement learning in cyber defense. Section 3 introduces the game model of the red and blue teams and the main idea of the proposed Node2vec-PPO algorithm. Section 4 introduces the construction of a high-fidelity, multi-action, fine-grained simulation environment. Section 5 presents a series of experiments conducted to evaluate the effectiveness of our method and analyzes the results. Finally, Section 6 concludes this paper.

## 2. Related Work

### 2.1. Autonomous Cyber Defense

Autonomous cyber defense, as a technology capable of adaptively perceiving, analyzing, and responding to cyber threats, aims to reduce human intervention and achieve intelligent and autonomous cyber defense [4]. The literature [5] emphasized that autonomous defense systems must possess adaptive capabilities and real-time responsiveness, with artificial intelligence and machine learning serving as the core supporting technologies. Early research on autonomous cyber defense focused on the application of deep learning and reinforcement learning in anomaly detection and optimization strategies. For example, the literature [6] developed an SVM-based intrusion detection system, and the literature [5] proposed using supervised learning models to classify network traffic. By training on datasets, the models can recognize known attack patterns. Although these methods can detect known attack patterns, they lack the capability to adapt strategies dynamically in evolving environments. With increasing sophistication of cyber attacks, dynamic defense mechanisms driven by artificial intelligence have attracted significant research attention. Recent advances include the development of deep reinforcement learning (DRL)-based frameworks that autonomously optimize defense strategies through environmental interactions [7]. Experimental validations have revealed that multi-agent reinforcement learning architectures can effectively coordinate defense operations in complex network scenarios [8]. In anomaly detection domains, deep learning architectures that incorporate autoencoders and Deep Neural Networks (DNNs) have demonstrated superior performance in processing high-dimensional traffic data compared to traditional methods [9]. However, current research paradigms predominantly neglect the critical influence of network topology on defense decision processes, resulting in suboptimal environmental perception during policy optimization cycles.

Game theory provides theoretical support for autonomous defense by modeling the strategies and payoffs of both the attacker and the defender. Early theoretical investigations established zero-sum game formulations that quantify static equilibrium states in cyber conflict scenarios [10]. Subsequent research extended these frameworks through Stackelberg game paradigms that dynamically adjust defense configurations by analyzing adversarial behavioral patterns [11]. Although multistage game approaches have been implemented for the prediction of critical node vulnerability [12], existing implementations predominantly employ discrete state representations that inadequately characterize network environmental continuity. A recent breakthrough is the WoLF-BSS-Q algorithm proposed by [13], which uses Bayesian inference to address uncertainties in attack strategies. The literature [14] constructed a stackelberg supergame model of cyberspace conflict to form a dynamic autonomous defense strategy. However, its computational efficiency in complex topological environments remains to be improved.

**Figure 1:**
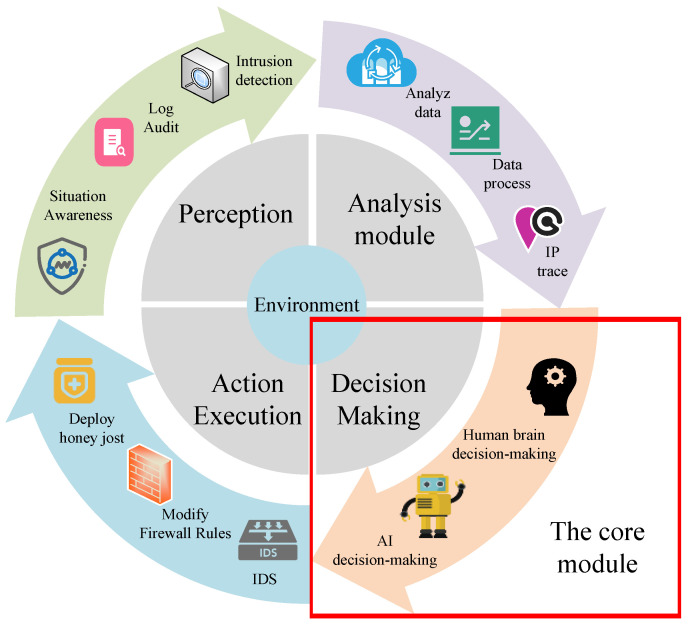
The framework of an autonomous cyber defense agent.

Autonomous cyber defense technologies, by integrating artificial intelligence, game theory, and optimization algorithms, have demonstrated significant potential to address complex and dynamic cyber attack scenarios. As shown in Figure 1, the basic framework of autonomous cyber defense is focused on the decision-making module, which is crucial to enabling agents to make quick and effective decisions. To achieve intelligent and autonomous cyber defense, scholars have begun integrating reinforcement learning with game theory to further optimize autonomous cyber defense strategies, thereby promoting the development of autonomous cyber defense technologies.

### 2.2. Reinforcement Learning in Cyber Defense

Reinforcement Learning (RL) has emerged as a promising paradigm for autonomous defense systems due to its inherent capacity for policy optimization through environmental interactions, particularly effective in dynamic operational contexts with evolving threat vectors. Recent studies have demonstrated the applicability of deep reinforcement learning architectures in cloud-edge defensive operations, achieving continuous decision-making cycles through environmental feedback [15]. However, conventional feature encoding schemes employed in these implementations exhibit limited capability in combining nodal attributes with topological connectivity patterns. Empirical evaluations of security alert-driven policy optimization frameworks reveal superior generalization characteristics compared to rule-based systems [16], although their action space designs remain limited to predefined atomic defense operations, impeding fine-grained response mechanisms. In particular, while adaptive DRL frameworks have been engineered to accommodate multistage adversarial campaigns [17], their practical implementation faces scalability challenges as the size of state space increases exponentially with network size, inducing significant degradation–degradation of training efficiency. Contemporary adversarial simulation platforms leveraging DRL architectures [18] demonstrate adaptive learning capabilities in uncertain cyber security environments, yet fundamentally lack operational awareness of complex network characteristics. Hierarchical DRL implementations for multi-strategy defense coordination [19] currently operate under oversimplified environmental assumptions, neglecting critical nodal attribute correlations in heterogeneous networks. Advanced permutation-invariant deep Q networks with set function encoding [20] show promise in multi-network threat mitigation scenarios but suffer from observational sparsity when processing low-frequency alert patterns, leading to suboptimal policy convergence.

RL methods can dynamically adjust defense strategies in response to changes in the network environment, thus enhancing their adaptability to evolving cyber threats and diverse network conditions. However, these methodological limitations collectively underscore the challenge inherent in contemporary RL-based defense systems: the intrinsic sparsity of discrete encoding representations, which impedes the accurate modeling of continuous network dynamics. Therefore, exploring more advanced embedding techniques and strategy optimization mechanisms is essential to improve the effectiveness and adaptability of autonomous cyber defense systems.

### 2.3. Simulation Environment

DARPA has launched a competition to create an automatic defense system that can independently adopt defense strategies and deploy them to actual networks [21]. To achieve this goal, we need to train our agents in a simulated environment. Through diverse training scenarios, agents can learn and train in a broader range of environments, improving their generalization capabilities and the scope of the application. OpenAI Gym [22] and PettingZoo [23] are currently the most widely used agent training and testing environments, offering benchmark collections and open source software libraries for training, testing, validating, and evaluating deep reinforcement learning algorithms. DARPA launched the Cyber Grand Challenge, which is a competition to create an automatic defense system that can detect flaws, develop patches, and deploy them in real time to the network.

Contemporary research in autonomous cyber defense systems has witnessed substantial progress in agent training frameworks. The NASim platform establishes the foundational infrastructure for penetration testing simulations through discrete network state representations [24], although its operational efficacy diminishes in large-scale deployments due to combinatorial state space explosion phenomena. While Microsoft’s CyberBattleSim introduces OpenAI Gym-compatible abstractions for reinforcement learning applications [25], its defensive action space remains constrained to legacy security mechanisms such as firewall configurations, failing to incorporate adaptive defense paradigms. Notable advancements emerge in military-grade simulation environments like CybORG [26], which implements multi-agent adversarial modeling through red/blue/white team architectures. This framework enables virtualized network replication via API-mediated environment synchronization, though restricted code accessibility impedes independent verification and methodological extensions. Parallel developments include CyGIL’s stateless architecture integrating MITRE ATT&CK frameworks for high-fidelity threat emulation [27], but similar transparency limitations hinder community-driven enhancement. Recent hybrid approaches demonstrate practical integration potentials, exemplified by PenGym’s fusion of the NASim, CyRIS, and Metasploit toolchains [28]. This platform enables concrete penetration testing actions in bounded network contexts through deterministic action modeling, though its defensive scope remains anchored in conventional security protocols. In contrast, Cyberwheel’s decoy deployment strategies introduce novel active defense mechanisms through high-level network abstractions [29], albeit lacking multi-agent coordination capabilities essential for modern threat landscapes.

However, as pointed out in previous works, such as [30,31], most existing agent training and validation environments only support abstract simulations of network spaces, lacking fine-grained simulations of real-world environments to improve training realism and efficiency. Therefore, constructing training environments with characteristics, fine-grained descriptions of network topologies, and support for multi-agent training algorithms is crucial for research on autonomous defense decision-making in cyberspace.

## 3. Methodology

In this section, we abstracted the network attack and defense processes as an incomplete-information non-cooperative game, constructing a game model between attackers and defenders to assist defenders in making optimal strategy choices under limited resources. To address the representation bottleneck of traditional methods in high-dimensional sparse environments, the Node2vec model is employed to encode network topology and security attributes into low-dimensional vectors. Section 3.1 introduces the construction of the game model. Section 3.2 proposes the autonomous cyber defense decision-making method based on graph embeddings.

### 3.1. Construction of the Game Model

By simulating attack and defense confrontations in cyberspace, this work achieves autonomous cyber defense. Our work models the attackers and defenders within the network as a typical dynamic non-cooperative game under conditions of incomplete information. To this end, this paper has expanded the trio in autonomous cyber defense: participants, participant strategies, and participant utilities. We have rationally abstracted key elements such as the autonomous cyber defense space, participant actions, and participant information, which can be represented as follows:

(1) $$G=\langle N,S,A,\Omega ,O,U\rangle$$

In the context of autonomous cyber defense, let *N* denote the set of participants; *S* represent the set of state spaces within the autonomous cyber environment; *A* denote the set of actions available to these participants; Ω represent the set of strategies employed; *O* denote the collection of information accessible to them; and *U* represent the set of utilities assigned to the participants.

#### 3.1.1. Game Participants and Space State Definitions

In the game decision-making of autonomous cyber defense, the participants of the game are attackers and defenders, which can be expressed as follows:

(2) $$N=\left\{n_{att}^{1},n_{att}^{2},\ldots ,n_{att}^{i}\right\}\cup \left\{n_{def}^{1},n_{def}^{2},\ldots ,n_{def}^{j}\right\}$$

where $n_{att}^{i}$ represents the *i*-th attacker, $n_{def}^{j}$ represents the *j*-th defender, and there is at least one attacker and one defender among the participants. To simplify the model and verify the effectiveness of our method, this paper considers a single attacker and a single defender: $N=\left\{n_{att}\right\}\cup \left\{n_{def}\right\}$.

**Figure 2:**
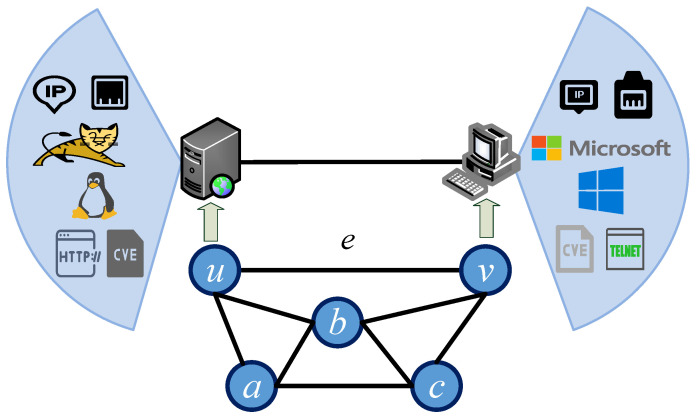
The state space of the game.

In the intelligent game of autonomous cyber defense, the state space of the game is the environment where cyber offense and defense confrontation is implemented, for example, in the network environment shown in Figure 2. This paper uses a graph model to represent the state space of the current game. This model includes various types of node sets and link sets within the network. The connections between nodes are represented using an adjacency matrix of the undirected graph, where 1 indicates a direct connection between nodes and 0 indicates no connection. Additionally, each node’s IP address, the operation adjacency matrix of the undirected graphing system, services, ports, and other security elements and attributes are included in the set. Therefore, we define the attributes information of the node as $T_{u}=\left\{ip,port,service,os,software,vul,\cdots \right\}$ and $T_{v}=\left\{ip,port,service,os,software,vul,\cdots \right\}$.

By integrating these elements, we can accurately represent the network’s topology and security characteristics through the graph model:

(3) S=<V,E,W,T>

where *V* represents the set of nodes in the state space, *E* represents the set of links in the state space, *W* represents the connection relationship matrix between nodes in the state space, and T represents the set of security elements and attributes contained in the nodes. Specifically, *V* enumerates individual nodes, T stores the attribute information for each node as a two-dimensional set, *E* explicitly lists pairwise connections for conceptual clarity, and *W* provides a matrix representation of the connection relationships for the machine to receive. In the game state shown in Figure 2: $V=\{V_{a},V_{b},V_{c},V_{u},V_{v}\}$; $E=\{E_{\left(a,u\right)},E_{\left(a,b\right)},E_{\left(a,c\right)},E_{\left(b,c\right)},E_{\left(b,v\right)},E_{\left(c,v\right)},E_{\left(u,b\right)},E_{\left(u,v\right)}\}$; $W=\left[\begin{array}{ccccc} 1 & 1 & 1 & 1 & 0\\ 1 & 1 & 1 & 1 & 1\\ 1 & 1 & 1 & 0 & 1\\ 1 & 1 & 0 & 1 & 1\\ 0 & 1 & 1 & 1 & 1 \end{array}\right]$;$T=\{T_{a},T_{b},T_{c},T_{u},T_{v}\}$.

#### 3.1.2. Action Spaces and Strategy Sets

In the intelligent game of autonomous cyber defense, according to the different types of participants, the action set of participants can be further divided into the action set of attackers and the action set of defenders, which can be expressed as follows:

(4) $$A=\left\{A_{att}^{1},A_{att}^{2},\ldots ,A_{att}^{i}\right\}\cup \left\{A_{def}^{1},A_{def}^{2},\ldots ,A_{def}^{j}\right\}$$

where $A_{att}^{i}=\left\{a_{i}^{1},a_{i}^{2},\cdots ,a_{i}^{n}\right\}$ represents the action set of the *i*-th attacker, containing *n* specific attack actions, and $A_{def}=\left\{a_{j}^{1},a_{j}^{2},\cdots ,a_{j}^{m}\right\}$ represents the action set of the *j*-th defender, containing *m* specific defense actions. Similarly, this paper constructs the environment as a single defender and a single attacker, so $A=\left\{A_{att}\right\}\cup \left\{A_{def}\right\}$. In modeling the actions of the red team (attacker), this study mapped the MITRE ATT&CK Kill Chain phases to Atomic Red Team (ART) techniques, thereby defining the red team action set [32]. This approach allows the ART agent to execute higher-level attack phases on the host, and the environment can cross-reference the target host’s attributes with the attributes of ART techniques. In modeling the actions of the blue team (defender), this paper considered proactive defense methods commonly used in current networks and defined the blue team’s action space, which includes actions such as deploying decoys, modifying firewall policies, and stopping services. These measures aim to slow down or prevent the red team’s attacks across the entire network. The collection of intelligent agents for both attackers and defenders is shown in Table 1 and Table 2.

In the intelligent game of autonomous cyber defense, according to the different types of participants, the strategy set of participants can be further divided into the strategy set of attackers and the strategy set of defenders, which can be expressed as follows:

(5) $$\Omega =\left\{\Omega_{att}^{1},\Omega_{att}^{2},\ldots ,\Omega_{att}^{i}\right\}\cup \left\{\Omega_{def}^{1},\Omega_{def}^{2},\ldots ,\Omega_{def}^{j}\right\}$$

where $\Omega_{att}^{i}=\left\{\omega_{i}^{1},\omega_{i}^{2},\cdots ,\omega_{i}^{n}\right\}$ represents the strategy set of the *i*-th attacker in state $S_{t}$, containing *n* specific attack strategies and $\Omega_{def}^{j}=\left\{\omega_{j}^{1},\omega_{j}^{2},\cdots ,\omega_{j}^{m}\right\}$ represents the strategy set of the *j*-th defender in state $S_{t}$, containing *m* specific defense strategies. This paper considers a single attacker and a single defender: $\Omega =\left\{\Omega_{att}\right\}\cup \left\{\Omega_{def}\right\}$. In the game model of autonomous cyber defense, the strategy refers to the action plans chosen by the blue team and the red team based on the current state of the network. The core of the game strategy lies in dynamically adjusting the configuration of defense devices or the selection of attack strategies based on the network topology, node attributes, and attacker behaviors. Attack strategies are the probability distributions of attack actions chosen by the red team based on the current network state (e.g., node exposure level and defense measures). For example, attack strategies may include scanning for vulnerabilities, lateral movement, and privilege escalation. Defense strategies are the probability distributions of defense actions chosen by the defensive agent based on the current network state (e.g., node vulnerabilities and attacker positions). For instance, defense strategies may include patching vulnerabilities, deploying firewall rules, and setting up decoy nodes. The defensive agent chooses the optimal strategy based on the current state (e.g., attacker positions and vulnerability exposure) and utility functions (e.g., the cost and benefit of patching vulnerabilities). For instance, if the attacker is close to key nodes, the defensive agent prioritizes modifying firewall rules; if the attacker’s behavior is covert, the defensive agent opts to deploy decoys for entrapment.

#### 3.1.3. Observation Space and Reward Shaping

In the intelligent game of autonomous cyber defense, according to the different types of participants, the information set of game participants can be further divided into the information set of attackers and the information set of defenders, which can be expressed as follows:

(6) $$O=\left\{O_{att}^{1},O_{att}^{2},\ldots ,O_{att}^{i}\right\}\cup \left\{O_{def}^{1},O_{def}^{2},\ldots ,O_{def}^{j}\right\}$$

where $O_{att}^{i}=\left\{o_{i}^{1},o_{i}^{2},\ldots ,o_{i}^{n}\right\}$ represents the *i*-th attacker information set, containing *n* individual attack information and $O_{def}^{j}=\left\{o_{j}^{1},o_{j}^{2},\ldots ,o_{j}^{m}\right\}$ represents the *j*-th defender information set, containing *m* specific defense information. Similarly, this paper considers $O=\left\{O_{att}\right\}\cup \left\{O_{def}\right\}$. The state of the game represents the general security status of the current network environment, including the network topology, the attributes of the nodes, and the behaviors of the attackers and defenders. This information is primarily represented through a graph model, which includes various types of node set and link set. The connections between nodes are represented using a matrix, where each node also includes attributes such as IP address, operating system, services, ports, and vuls. These game state information provide the basic structure and characteristics of the network. The detection results include the real-time monitoring and detected attack behaviors and abnormal traffic by the defense system. This information comes from various security devices on the network, such as firewalls, intrusion detection systems (IDSs), and intrusion prevention systems (IPSs). A detector layer is set up in the network to detect any red team operations, reflecting the current status of node information. The detection results are typically Boolean data. By combining the state of the game and the detection results, the defensive agent can more comprehensively understand the current network environment, enabling more precise and efficient defense decisions. This layered observation information helps to enhance the environmental perception capabilities of the defensive agent.

In the intelligent game of cyber offense and defense, according to the different types of participants, the utility set of game participants can be further divided into the utility set of attackers and the utility set of defenders, which can be expressed as follows:

(7) $$U=\left\{U_{att}^{1},U_{att}^{2},\ldots ,U_{att}^{i}\right\}\cup \left\{U_{def}^{1},U_{def}^{2},\ldots ,U_{def}^{j}\right\}$$

where $U_{att}^{i}$ represents the utility of the *i*-th attackers and $U_{def}^{j}$ represents utility of the *j*-th defenders and is constrained by the state space of the game, the actions, strategies, and information of both parties. Similarly, this paper considers $U=\left\{U_{att}\right\}\cup \left\{U_{def}\right\}$. As researchers of the autonomous cyber defense decision-making method, this paper considers the effectiveness of the designed method for autonomous defenders, so this paper mainly considers the utility of the blue intelligent agent. When taking actions in state $S_{t}$, the blue intelligent agent receives a reward $R_{t}$, which can be positive or negative. The negative value primarily arises because the defensive actions taken by the blue team may not immediately stop the attacker’s actions but will consume defense resources. For example, when deploying decoys, the red team’s target may not necessarily be the decoy host.

Since cyberspace is a typical human-made complex giant system, in the autonomous cyber defense decision-making framework based on intelligent games, the attackers and defenders are in a typical non-cooperative zero-sum game and have the characteristics of typical dynamic non-stationarity, incomplete information, and bounded rationality. These characteristics result in the state space of the offense–defense game constantly changing with the confrontation process. Attackers and defenders can only observe their own states and limited opponent states. When choosing strategies and executing actions, there is also a temporal sequence between attackers and defenders. Therefore, combining the intelligent game model, which encompasses dynamic non-stationarity, incomplete information, and bounded rationality, with the autonomous defense decision-making process, the specific mapping relationship is shown in Table 3.

The intelligent game-based autonomous defense decision-making process can be described as follows. Participants in the game space select the corresponding strategies, implement the corresponding actions, and obtain the corresponding utilities based on the information they acquire. Combined with Table 3, this intelligent game process can be expressed as follows:

(8) $$U\left(T^{k}\right)=\sum_{t=t_{o}}^{T^{k}}f_{u^{k}}\left(S,O^{k}|S,A^{k}{|}_{O^{k}}^{\Omega^{k}},t\right)$$

where $U\left(T^{k}\right)$ represents the utility obtained by a participant after $T^{k}$ rounds of the game and $f_{u^{k}}\left(S,O^{k}|S,A^{k}{|}_{O^{k}}^{\Omega^{k}},t\right)$ represents the utility function of the *k*-th participant. The state space *S*, the information $O^{k}|S$ of the participant in the game state space, the actions $A^{k}{|}_{O^{k}}^{\Omega^{k}}$ determined by the information and strategy, and the game round *t* jointly determine the form of the utility function. In this paper, we evaluate the utility of the blue team.

### 3.2. Node2vec-PPO Method

In network confrontations, characteristics such as unclear boundaries, variable rules, incomplete information, and irrational adversarial behavior exist. This requires that autonomous cyber defense agents perceive and make decisions efficiently in complex network topology environments. Therefore, it is crucial to effectively represent the key elements in the modeling process. Traditional simple encoding methods have significant limitations in high-dimensional, dynamic network environments. In large-scale networks with a large number of nodes, directly using node identifiers leads to an explosion of the feature dimension. For example, in a network containing 1000 nodes, using one-hot encoding results in an input dimension as high as 1000, but the effective information on inter-node associations is submerged in sparsity. Traditional methods fail to capture deep features of network topology, such as node importance, topological structures, and potential attack paths, making it difficult for agents to understand the attacker’s penetration logic.

To address these critical issues, this paper introduces a Node2vec graph embedding representation combined with the Proximal Policy Optimization (PPO) reinforcement learning algorithm, providing an innovative solution. The Node2vec model maps network nodes into a low-dimensional continuous vector space, achieving an efficient representation of network topology and security attributes. Through biased random walks (combining Breadth-First Search BFS and Depth-First Search DFS), Node2vec can simultaneously capture local neighborhood information, such as the vulnerabilities of single-hop nodes, as well as global structural features like hub nodes and attack paths. For example, a database server connected to multiple critical service nodes will have its embedding vector closer to other high-value nodes in the low-dimensional space. Additionally, Node2vec compresses high-dimensional sparse node security attributes into low-dimensional dense vectors, significantly reducing the complexity of model input. It can integrate static attributes such as vulnerability information from nodes and types of operating system with dynamic attributes such as alert states. The following introduces the algorithm principle.

#### 3.2.1. Node2vec Model in ACD

**Figure 3:**
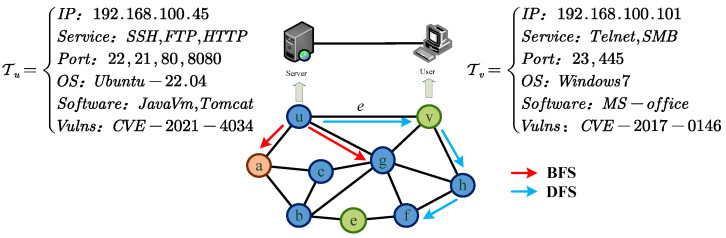
The search strategies of the Node2vec model.

Node2vec extends the definition of neighbors based on Deepwalk’s graph definition. For each vertex *u* in the network topology, $N_{s}\left(u\right)$ is defined as the set of neighbors of vertex *u* sampled by the sampling strategy *S*. We can regard the neighbor sampling problem of the source node as a form of local search, limiting the size of the neighbor set $N_{s}\left(u\right)$ to *t* nodes. There are two extreme sampling strategies for generating a set of *t* nodes: the first is BFS, where neighbors $N_{s}\left(u\right)$ are limited to the immediate neighbors of the source node *u*, as shown in Figure 3 with *a*, *v*, and *g*, where t=3. The second is DFS, where neighbors $N_{s}\left(u\right)$ consist of a sequence of nodes with increasing distance from the source node *u*, as shown in Figure 3 with *v*, *h*, *f*, *e*, *b*, and *c*. The neighbors obtained by the BFS strategy generally have structural similarity, while those obtained by the DFS strategy better meet the homogeneity of the network.

Based on the concepts of BFS and DFS, a flexible biased random walk strategy is designed to smoothly integrate BFS and DFS into this strategy. As shown in Figure 3, given the current vertex *v*, a random walk with a step length of *l* is to be performed. Let the next vertex visited be *x*, with $c_{i}$ representing the *i*-th node in the walk sequence and $c_{0}=v$. The transition probability from node *v* to the next vertex *x* is as follows:

(9) $$P\left(\left.c_{i}=x\right|c_{i-1}=v\right)=\left\{\begin{array}{c} \frac{\pi_{vx}}{Z}\;\;\;if\left(v,x\right)\epsilon E\\ 0\;\;\;\;\;\;otherwise \end{array}\right.$$

where $\pi_{vx}$ is the unnormalized transition probability between vertex *v* and vertex *x*, and *Z* is the normalization constant. Node2vec introduces two hyperparameters *p* and *q* to control the random walk strategy.

Assuming that the current random walk passes through edge e=(u,v) to reach vertex *v*, in determining the next walking direction from vertex *v*, it is necessary to evaluate the transition probabilities on edge $\left(v,x\right)$ and choose the edge with the highest transition probability as the next walking direction, that is, to decide whether the next vertex is *g* or *h*. Let $\pi_{vx}=\alpha_{pq}\left(u,x\right)\cdot w_{vx}$ and $w_{vx}$ be the weights of the edges between vertex *v* and vertices *x*. Respectively, let **$d_{ux}$** be the shortest path distances between the vertex *u* and vertices *x*. Then, the transfer probability is

(10) $$\alpha_{pq}\left(u,x\right)=\left\{\begin{array}{c} \frac{1}{p}\;if\;d_{ux}=0\\ 1\;\;if\;\;d_{ux}=1\\ \frac{1}{q}\;if\;\;d_{ux}=2 \end{array}\right.\;$$

The parameter *p* controls the probability of revisiting the just-visited vertex. Note that *p* only applies to the case where $d_{ux}=0$, which means that vertex *x* is the vertex that just visited before the current vertex *u*. If *p* is high, the probability of revisiting the just-visited vertex will be low, and vice versa. The parameter *q* controls whether the walk is outward or inward. If q>1, the random walk tends to visit vertices close to *u*, favoring BFS, which helps capture local neighborhood information and quickly identify direct vulnerabilities of the current node, such as open ports and unpatched vulnerabilities. If q<1, it tends to visit vertices far from *u*, favoring DFS, which helps to explore the global network structure. By using the DFS strategy, defenders can anticipate that attackers may penetrate core data through multi-hop nodes and discover potential cross-subnet attack paths.

Node2vec generates node sequences through biased random walks and learns low-dimensional embedding vectors using the Skip-gram model from natural language processing [33]. Node2vec embeds the network topology information obtained S=<V,E,W,T>, serving as one of the core components of the blue agent observation space in the game. After obtaining the Node2vec random walk sequence, the objective function to be optimized is defined as follows:

(11) $$max_{f}\sum_{u\in V}\log Pr\left(N_{s}\left(u\right)|f\left(u\right)\right)$$

where $f\left(u\right)$ is the mapping function that maps vertex *u* to the embedding vector. To make the objective function easier to handle, two assumptions are made: the conditional independence assumption and the feature space symmetry assumption. The conditional independence assumption means that given the source vertex *u*, the probability of its neighboring vertices occurring is independent of the other vertices in the neighbor set:

(12) $$Pr\left(N_{s}\left(u\right)|f\left(u\right)\right)=\prod_{n_{i}\in N_{s}\left(u\right)}Pr\left(n_{i}|f\left(u\right)\right)$$

The feature space symmetry assumption means that a vertex shares the same embedding vector when it acts as both a source vertex and a neighboring vertex. Under this assumption, the conditional probability formula can be expressed as follows:

(13) $$Pr\left(n_{i}|f\left(u\right)\right)=\frac{exp(f\left(n_{i}\right)\cdot f\left(u\right))}{\sum_{v\in V}exp\left(f\left(v\right)\cdot f\left(u\right)\right)}$$

According to the above two assumptions, the final objective function is expressed as follows:

(14) $$max_{f}\sum_{u\in V}\left[-\log Z_{u}+\sum_{n_{i}\in N_{s}\left(u\right)}f\left(n_{i}\right)\cdot f\left(u\right)\right]$$

Since the computation cost of the normalization factor $Z_{u}=\sum_{n_{i}\in N_{s}\left(ut\right)}exp\left(f\left(n_{i}\right)\cdot f\left(u\right)\right)$ is high, negative sampling is used for optimization. Specifically, when dealing with large-scale datasets, the negative sampling technique accelerates the model training process by randomly selecting a portion of the samples as negative samples instead of calculating all samples. The stochastic gradient descent algorithm is used to optimize this objective function, and node embeddings are obtained during the optimization process.

#### 3.2.2. Node2vec-PPO Algorithm

In the study of autonomous cyber defense decision-making, we select the PPO reinforcement learning algorithm as our core methodology. Compared to traditional policy gradient methods, PPO mitigates the issues of policy oscillation and low sample efficiency inherent in those algorithms. It does so by introducing policy update step constraints and objective function clipping techniques. It significantly enhances sample collection efficiency through multi-threaded parallel environment sampling. This feature is particularly critical for dynamic and uncertain cyber attack–defense scenarios. For details on PPO, see the work of Schulman [34]. Our design for the observation space of the defensive agent consists of two parts: one is the boolean alerts information generated by the original attacker behaviors, and the other part is the node embeddings generated by Node2vec representing the network topology. After representing the network topology information using the Node2vec model, we input the resulting embeddings into the policy network of the PPO algorithm, along with the alerts generated in the network environment. These combined inputs form the observation of the agent, expressed as O=[alertinfo,Node2vec(S)]. The core of the Node2vec-PPO algorithm lies in optimizing the following policy objective function:

(15) $$\begin{array}{c} L_{\pi }\left(O,a\right)=\min\left(\frac{\pi \left(a|O\right)}{\pi_{old}\left(a|O\right)}A^{\pi_{old}}\left(O,a\right),\right.\\ \left.clip\left(\frac{\pi \left(a|O\right)}{\pi_{old}\left(a|O\right)},1-\epsilon ,1+\epsilon \right)A^{\pi_{old}}\left(O,a\right)\right) \end{array}$$

where $\pi \left(a|O\right)$ is the current policy, $\pi_{old}\left(a|O\right)$ is the policy from the previous iteration, $A^{\pi_{old}}\left(O,a\right)$ is the advantage function representing the advantage of taking action *a* in state *s* under the current policy $\pi_{old}$, and ϵ is a small positive number used to limit the step size of policy updates. The clip function restricts the clipping of the probability ratio, which is the ratio of the action probabilities generated by the old policy and the new policy. This ratio is constrained within a range to prevent the policy from making too large changes during updates. In autonomous cyber defense scenarios, PPO’s policy iteration stability enables effective responses to dynamic environmental changes and uncertainties in attack behaviors. The general Node2vec-PPO algorithm is shown as Algorithm 1. This design allows encoded vectors to represent common features of different networks, including node attributes and structural information in the network topology, reflecting dynamic changes in the network state such as vulnerability patching and node isolation. The embeddings generated by Node2vec and the alert information are the core components of the game state space $S=\langle V,\;\;E,\;\;W,T\rangle$, directly influencing the choice of defense strategies. For each game state, when node attributes change, such as vulnerability patching or service shutdown, running Node2vec can update the embeddings in real time to reflect the network state.

**Figure 4:**
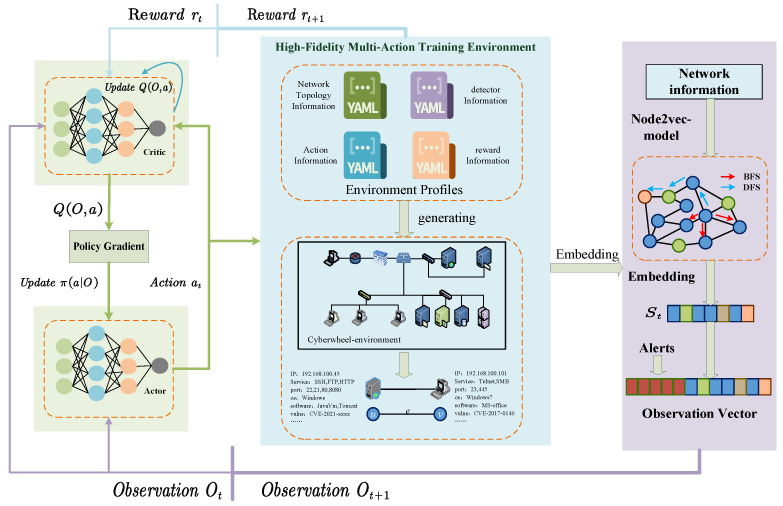
The framework of Node2vec-PPO.

The structure of the proposed method is shown in Figure 4. We validated our method in the Cyberwheel environment introduced in Section 4. In this environment, detectors are designed, and each red action is associated with a set of techniques to simulate how the actions are executed in the real world. The detectors on each node check the techniques used by the red actions and compare them with their own set of techniques. For each technique used by the red actions in the detector set, the detector may create an alert for the interaction, and the generated alert information is composed of one-hot boolean data. In addition to the alert information generated by detecting red-side operations in the network, the entire network is also represented in the form of Node2vec as the observation input for the agent and trained using the PPO algorithm. This method can improve the perception capabilities of the agent and further improve its autonomous decision-making abilities.
**Algorithm 1** Node2vec-PPO Algorithm.**Input:** 
Environment env, policy network parameter θ, value network parameter ϕ, experience replay buffer *D*, tailoring range hyperparameter ε, batch size *M*, round length *T*, optimization steps *k*, Node2vec parameters**Output:** 
Optimal strategy π   1:**for** episode ≠ max_episodes **do**   2:    Reset environment $S_{0}∼env$   3:    Initialize experience replay buffer *D*   4:    **for** each step in episode **do**   5:        Set $S_{t}∼S_{0}$   6:        Generate node embeddings using Node2vec   7:        Extract boolean alert information   8:        Form observation $O_{t}=\left[\mathrm{alert}\;\mathrm{info},\mathrm{Node}2\mathrm{vec}\left(S_{t}\right)\right]$   9:        Select action $A_{t}∼\pi_{\theta }\left(O_{t}\right)$10:        Execute $A_{t}$, receive $R_{t}$, observe $S_{t+1}$11:        Store $\left(O_{t},A_{t},S_{t+1},R_{t}\right)$ in *D*12:        **if** episode terminates or length *T* is reached **then**13:             Compute advantage ${\hat{A}}_{t}$ and return $G_{t}$14:             Estimate value $V_{\phi }\left(O_{t}\right)$15:             ${\hat{A}}_{t}=G_{t}-V_{\phi }\left(O_{t}\right)$16:             Sample a batch of size *M* from *D*17:             **for** k=1 to *K* **do**18:                 Compute $r_{t}\left(\theta \right)=\pi \left(a|s\right)/\pi_{old}\left(a|s\right)$19:                 Compute $L_{\pi }\left(s,a\right)$ using clipping range ϵ20:                 Update θ by minimizing the loss21:                 Update ϕ using gradient descent22:             **end for**23:             Save Optimal strategy π24:             Clear buffer *D*25:        **end if**26:    **end for**27:**end for**

## 4. Construction of a High-Fidelity Multi-Action Training Environment

To validate the effectiveness of our graph embedding-based autonomous defense decision-making method, we require a simulation environment that supports complex network topology modeling, fine-grained attack-defense interactions, and dynamic state updates. Among the various environments introduced in Section 2.3, this study ultimately chose Cyberwheel for several reasons:

Firstly, Cyberwheel’s open-source nature meets our development needs, allowing us to customize the environment extensively. Secondly, the network in Cyberwheel comprises routers, subnets, and hosts, aligning with our requirement for a fine-grained, high-fidelity training environment. Routers manage network traffic between subnets; subnets represent broadcast domains and manage traffic between hosts; and hosts—belonging to subnets—contain lists of running services, ports, CVE vulnerabilities, and other security attributes. Moreover, the environment builds network information through flexible YAML file configuration, facilitating modifications to the network topology to meet the demands of our network scenarios. The configuration files define detectors, where each non-decoy host has a corresponding boolean value indicating whether it has been detected by the detector, generating alerts in the agent’s observation information. In the Cyberwheel environment, for the red team’s technology to successfully run killchain attacks on a host, the following conditions must be met: The target host’s operating system is included in the supported platforms; the technology includes the killchain phase in its supported killchain stages; the technique can exploit any CVE present on the target host; and the red team selects actions based on the attack chain and attack techniques, such as performing scans, exploiting vulnerabilities, escalating privileges, or causing impacts. This fine-grained attack logic makes the attack behaviors more realistic. Cyberwheel’s native support for red–blue confrontation makes it the ideal foundational platform to meet the above requirements. Using this environment allows one to realistically simulate attack–defense confrontations in complex network settings, providing robust support for validating our proposed autonomous defense decision-making method.

To meet the needs of research on autonomous cyber defense, this work has deeply modified and extended the Cyberwheel environment. Firstly, based on the original YAML configuration files, this paper refined the descriptions of network topology and node attributes, adding security-related fields such as firewall rules and vulnerability information, and improved the firewall settings within the network topology. This ensures that attackers are filtered during scanning instead of directly obtaining all nodes in other subnets. The modified network topology supports more fine-grained attack-defense modeling, increasing the complexity and realism of the experiments and making the experimental results more practically valuable.

To achieve real-time representation learning of the network topology, this paper has deeply integrated the Node2vec module into Cyberwheel. Based on dynamic changes in the network topology, we extract the node set *V*, edge set *E*, connection matrix *W*, and node attributes T from Cyberwheel’s YAML configuration, using NetworkX to generate a weighted graph $G=\left(V,E,W\right)$, with node attributes T as additional features. Regardless of the state of the network changes in each episode, Node2vec is automatically triggered for recalculation. The embeddings generated by Node2vec are concatenated with Cyberwheel’s original alert information as input to the PPO policy network. In this way, the defensive agent perceives the dynamic changes in the network environment more effectively, enhancing the precision and adaptability of its decision-making.

Furthermore, this study has added two types of fine-grained defensive actions for the blue team (defender) and implemented their interaction logic with the network state. The first action is the dynamic modification of the firewall rules, allowing the defender to add firewall rules to restrict the attacker’s lateral movement, which is shown in Algorithm 2. The second action is vulnerability patching, as shown in Algorithm 3, where the defender can fix specific vulnerabilities (CVE numbers) to eliminate attack points exploited by the attacker. After executing these actions, the state space of the game *S* is updated by modifying node attributes such as vulnerabilities and firewall_rules. Through these modifications to the Cyberwheel environment, we can more realistically simulate the decision-making process and utility performance of defensive agents when faced with complex network attacks, providing a high-fidelity experimental platform for research in autonomous cyber defense.
**Algorithm 2** Modify firewall rules function.**Input:** 
network, decoy_list, and alert**Output:** 
BlueActionReturn   1:action←alert.action   2:attacker_ip←alert.src_host   3:target_hosts←alert.target_host   4:valid_hosts←get_valid_hosts(target_hosts)   5:**if** valid_hosts is empty **then**   6:    **return** BlueActionReturn(False)   7:**end if**   8:**for** each host in valid_hosts **do**   9:    add_modify_firewall(host,action,attacker_ip)10:**end for**11:**return** BlueActionReturn(True)

**Algorithm 3** Patch vulnerability function.
**Input:** 
network, host, patches, applied_patches**Output:** 
BlueActionReturn   1:os←host.os; vulns←host.vulnerabilities   2:**for** each vuln in vulns **do**   3:    **if** patch.os=os **and** patch.cve=vuln **then**   4:         host.apply_patch(patch)   5:         host.remove(vuln)   6:         **break**   7:    **end if**   8:
**end for**
   9:**if** any patch was applied **then**10:    Append applied patches to applied_patches11:    **return** BlueActionReturn(True)12:
**else**
13:    **return** BlueActionReturn(False)14:
**end if**



## 5. Experiment

The experiments were conducted in the Cyberwhell simulation environment. Section 5.1 describes the scenario, after which we conducted a series of experiments. Section 5.2 analyzes the results of the experiments.

### 5.1. Experimental Scenario

**Figure 5:**
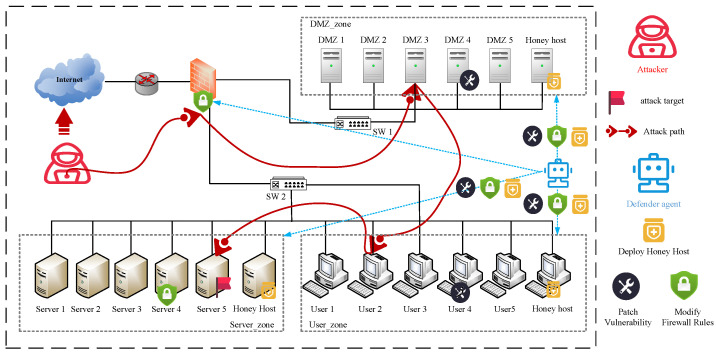
Experimental topology.

To verify the effectiveness of our proposed method, this paper abstracts the real environment shown in Figure 5 into a highly simulated environment provided by Cyberwheel for experiments. We chose to integrate autonomous cyber defense strategies into the information of 15 nodes (no honeypot in the initial situation) for a series of experiments, as shown in Figure 5. This network topology consists of a network of 15 hosts, 3 subnets, and 1 router. To test the effectiveness of the autonomous cyber defense agent, this paper used the Node2vec-PPO algorithm. The number of training episodes is 1000, and each episode consists of 100 steps. It is also ensured that the parameters of the PPO are kept consistent during the experiment. In Table 4, the environment hyperparameter settings are shown.

### 5.2. Experimental Analysis

**Figure 6:**
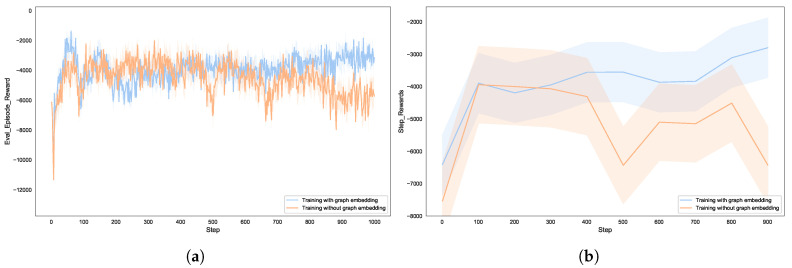
The effects of with or without graph embedding on the utility of autonomous defense agent: (**a**) reward for each episode; (**b**) reward for steps.

This paper tested the behavior of the blue agent in two experimental scenarios: one where only boolean types were used to represent the network information, and the other where Node2vec was used to represent important information about the network topology, which was then combined with alarm information generated by the detectors and included in the agent’s observation vector for testing. Figure 6 shows the expected utility of the autonomous defense agent. When the blue agent takes the corresponding actions to delay the red agent by several steps, it gains marginal positive rewards. However, regardless of the blue agent’s actions, while it can delay the red agent’s actions, the red agent will eventually continue to attack the server, resulting in a negative final reward.

When only boolean alert information is used as input to the agent, the information received by the agent is abstract and incomplete. Although the agent can make decisions, these decisions are not necessarily optimal. When Node2vec is used to further represent network topology information, the agent’s perception of the environment is enhanced, allowing it to choose better strategies to obstruct the attacker’s actions. It learns to always choose defense strategies to effectively stop the red agent. The results show that the utility performance of the blue team improves under the condition of graph representation, validating the effectiveness of our game model. In training with graph embeddings, the model shows a stable improvement after the training begins, while the model without graph embeddings remains highly volatile until later stages. This indicates that graph embeddings capture structural features of the network topology (such as node dependencies and attack paths), providing the agent with more efficient decision-making criteria. The fluctuation range of the curve using graph embeddings is significantly smaller than that of the curve without graph embeddings, suggesting that graph embeddings, through low-dimensional dense representations, reduce noise interference in the state space and enhance the stability of policy updates. Traditional one-hot encoding deals with sparse features, whereas our Node2vec-PPO method encodes security attributes of nodes, such as vulnerability states and firewall rules into embedding vectors. This allows the agent to perceive changes in the state of the network in real time, providing high-density information input for the PPO algorithm to dynamically adjust defense strategies.

To further explore the impact of Node2vec parameters on autonomous defense decision-making, we conducted multiple experiments to analyze the performance of the blue team under different parameter settings. The settings of the specific parameter are shown in Table 5.

**Figure 7:**
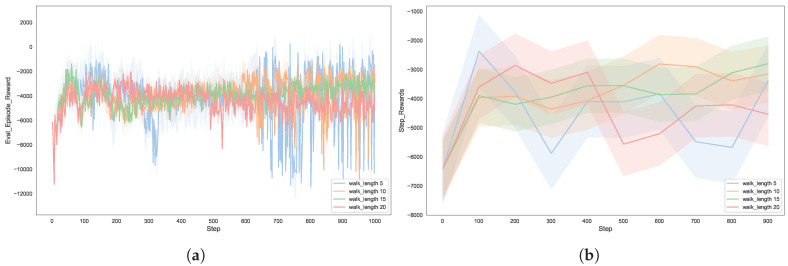
The effects of embedding processes with different walk lengths on autonomous defense agent: (**a**) reward for each episode; (**b**) reward for steps.

The number of walks determines the sampling depth of the Node2vec algorithm when generating node sequences. As shown in Figure 7, this paper conducted experiments with sampling depths of 5, 10, 15, and 20. The experimental results indicate that when the number of walks is low, the sampling of node sequences is insufficient, resulting in incomplete graph representation information, which adversely affects the decision-making ability and stability of the agent. As the number of walks increases, the sampling of node sequences becomes more adequate, leading to richer graph representation information and significantly improved decision-making ability of the agent. However, when the number of walks reaches a certain threshold, the improvement in utility levels off, indicating that an excessive number of walks does not bring significant performance gains and may instead increase computational overhead.

**Figure 8:**
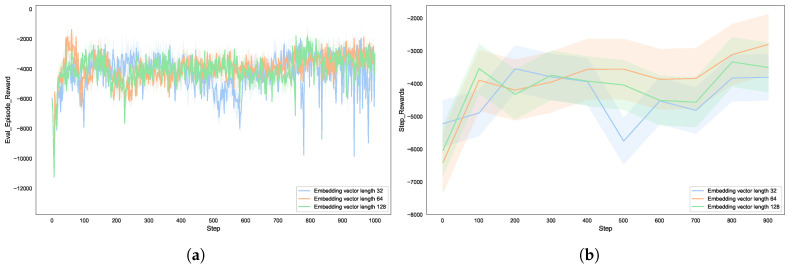
The effect of different embedding vector lengths on autonomous defense agent: (**a**) reward for each episode; (**b**) reward for steps.

The dimension of the node representation determines the dimensions of the embedding vectors generated by Node2vec. As shown in Figure 8, this study conducted experiments with each node having embedding lengths of 32, 64, and 128, with a fixed walk length (walk_length = 15). The experimental results show that in the initial stages of training, the cumulative reward for low-dimensional embeddings is significantly higher than that for high-dimensional embeddings, indicating that low-dimensional vectors accelerate the preliminary exploration of the policy network by simplifying the feature space. However, as the number of training steps increases, the performance improvement of low-dimensional embeddings gradually stagnates, indicating that low-dimensional embeddings lose information and fail to capture the global features of complex topologies.

Despite the fact that high-dimensional embeddings initially converge more slowly, they show significant advantages in the later stages of training, with final rewards steadily increasing. High-dimensional embeddings retain more structural details, assisting the agent in formulating better strategies in complex attack chains. Therefore, as the node representation dimension increases, the effectiveness of the strategy (blue team’s utility) gradually improves. However, when the dimension reaches a certain value, the efficiency improvement rate decreases. The lower dimensions do not fully express the complex structure of the network topology, making it difficult for the agent to accurately identify key nodes and attack paths. As the dimensions increase, the embedding vectors better capture the structural information of the network, significantly enhancing the agent’s decision-making ability. However, when dimensions exceed a certain threshold, the improvement in utility tends to plateau, indicating that excessively high dimensions do not bring significant performance gains and may instead increase the computational complexity of the model.

**Figure 9:**
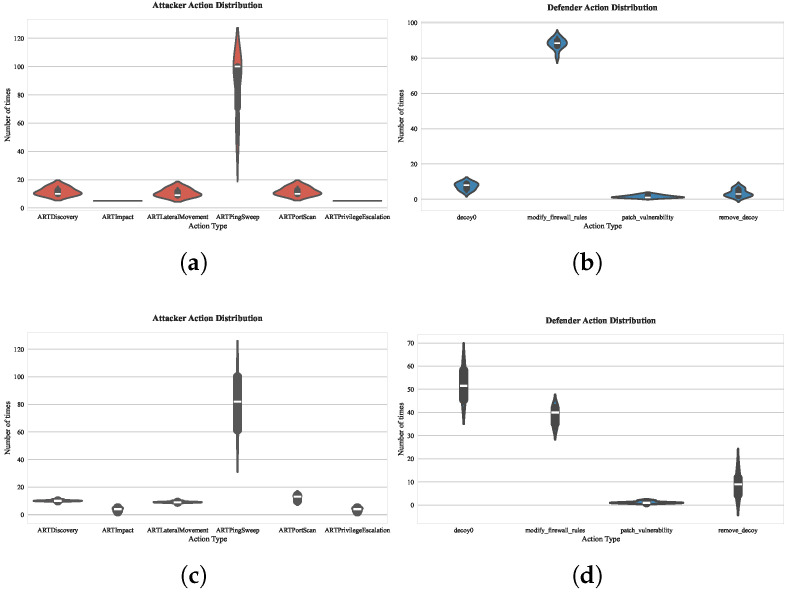
Distribution statistics of actions in offensive and defensive strategies under different *q* parameter strategies: (**a**) 1000-episode evaluation of attacker action distribution under parameter p=1,q=0.5; (**b**) 1000-episode evaluation of defender action distribution under parameter p=1, q=0.5; (**c**) 1000-episode evaluation of attacker action distribution under parameter p=1,q=2; (**d**) 1000-episode evaluation of defender action distribution under parameter p=1,q=2.

To fully understand the driving mechanism of the parameters *p* and *q* in Node2vec on the selection of the red and blue sides strategy, firstly, we discuss the in–out parameter, *q*, which controls the direction of exploration, whether it is an inward search or an outward search. As shown in Figure 9, by comparing the action distribution of the red and blue sides under the two sets of parameters p=1,q=0.5 (DFS tendency) and p=1,q=2 (BFS tendency), the embedding vectors generated by DFS can identify the key paths across subnets, driving the defender to prioritize reinforcement of hub nodes and modify the firewall policies of the target hosts. While the BFS strategy focuses more on local exposure points, the defender tends to respond quickly to surface threats, that is, to deploy decoys in the current subnet environment to hinder the attacker’s attack stage as much as possible. In complex attack scenarios, the embedding vectors of the DFS strategy can enable the defender to identify possible attack paths and concentrate resources to protect multiple target nodes. The BFS strategy is suitable for quickly responding to surface threats and deploying decoys within the target subnet to impede the attacker’s attack. As for why the BFS strategy for vulnerability repair is chosen to be less, it is actually because the defender does not know which vulnerability the attacker is exploiting. Choosing to repair vulnerabilities during training interaction cannot effectively hinder the attacker’s behavior, which also corresponds to the actual network scenario.

**Figure 10:**
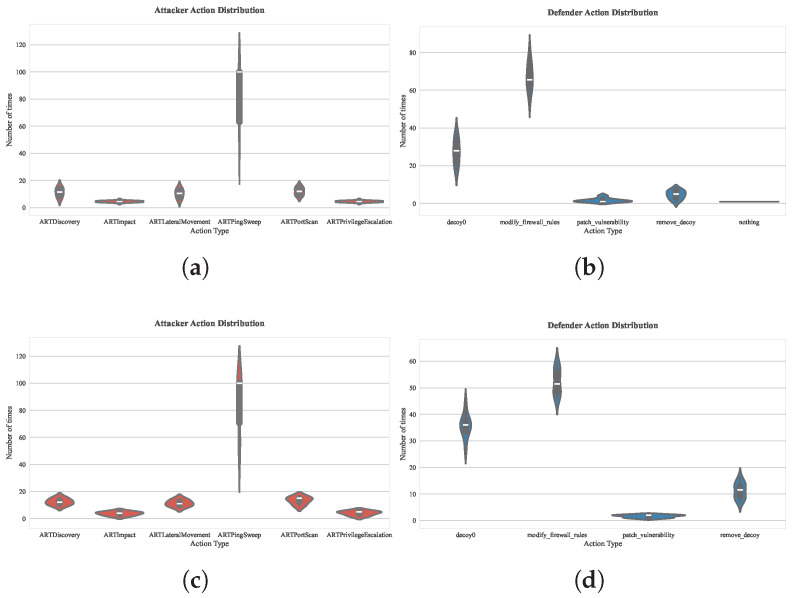
Distribution statistics of actions in offensive and defensive strategies under different *p* parameters: (**a**) 1000-episode evaluation of attacker action distribution under parameter p=0.5,q=1; (**b**) 1000-episode evaluation of defender action distribution under parameter p=0.5,q=1; (**c**) 1000-episode evaluation of attacker action distribution under parameter p=2,q=1; (**d**) 1000-episode evaluation of defender action distribution under parameter p=2,q=1.

Secondly, the related parameter is the return parameter, *p*, which can control the probability of revisiting the previously visited vertex. We also verified this, as shown in Figure 10. By comparing the action distribution under the two sets of parameters p=0.5,q=1 (exploratory walk) and p=2,q=1 (conservative walk), the defender’s actions in the exploratory walk are concentrated on patch_vulnerability and modify firewall_rules; while in the conservative walk, the defender’s actions are mainly basic decoy deployment. The embedding vectors generated by the exploratory walk can capture more potential attack paths, requiring the defender to prioritize repair of high-threat vulnerabilities and dynamically adjust the rules of the firewall. The conservative walk leads embedding vectors to focus overly on exposed local nodes, and the defender tends to deploy low-cost decoys rather than deep reinforcement.

## 6. Conclusions and Future Work

In this work, we successfully constructed an autonomous cyber defense agent capable of dynamically adjusting defense strategies by combining the Node2vec graph embedding method with game theory. Firstly, this study constructed a game model for both the red and blue teams. Then, the Node2vec model is applied to represent the network topology environment, mapping the node configuration information and the network topology structures to a low-dimensional vector space. Combined with the alert information from the network, this formed the observation vector for the agent, which is trained using the PPO algorithm for the blue agent. Secondly, in the training environment, this paper mainly added two types of action spaces, modifying firewall rules and fixing vulnerabilities, which better align with the needs of real cyber defense scenarios. The experimental results show that the method proposed in this paper can effectively improve the defense effectiveness of the blue team in complex network environments. Through an experimental analysis of the relevant parameters of the Node2vec model, this paper further verifies the impact of walk length and node representation dimensions, as well as the adoption of a sampling strategy on autonomous defense decision-making.

In this paper, the Node2vec-PPO approach we employ focuses primarily on network topology. However, when applied to large-scale complex networks, this real-time encoding method demonstrates inefficiency. Additionally, in networks with an excessive number of nodes and vast amounts of information, our representation may encounter the Curse of Dimensionality. Future research could explore graph representation methods such as Graph Neural Networks (GNNs) to enhance the agent’s perception capabilities. Future work should involve interfacing action modules and developing a more granular simulation environment to improve the transferability of autonomous cyber defense agents, thereby enabling the direct deployment of trained agents into real-world network environments. 

## Figures and Tables

**Table 1:** Red agent action space.

ATT&CK Kill Chain	Red Agent Action Space Description
Gathering information	Collecting vulnerability information of the target network, including network scanning, port service scanning, vulnerability scanning, and social engineering-based information gathering techniques.
Vulnerability Discovery	Discovering exploitable vulnerable ports.
Exploitation Launching Attacks	Using existing attack payloads or self-made malware.
Privilege Escalation	Escalating the operating privileges of the target host.
Lateral Movement	Continuing to scan and probe within the internal network, obtaining the network topology and the next exploitable host.
Attack Execution	Controlling the target host, stealing confidential information, uninstalling important software, and disrupting normal operation of the system.
No Action	Monitoring the current network status.

**Table 2:** Blue agent action space.

Defense Phase	Blue Agent Action Space Description
Monitoring	Gathering information, with alert information on the host to indicate whether the current node has an attacker intelligent agent attack, and obtaining alert information from internal network hosts.
Decoy Deployment	Deploying honeypot decoy nodes to trap attackers and hinder their attacks.
No Action	Maintaining the current network state.
Modifying Firewall Rules	Adding firewall rules to specific target hosts to mitigate or block further attacks from the attacker.
Vulnerability Repair	Repairing vulnerabilities on the target host, using existing patches to repair the target host to prevent further penetration by the attacker.

**Table 3:** The mapping relationship between autonomous cyber defense decision-making and game models and the reinforcement learning paradigm.

Autonomous Defense Cyber Decision-Making	Game Model	Reinforcement Learning
Defense Decision State Space	Game State Space	Interactive Environment Space
Defense Decision-Maker Set	Participant Set	Agent Set
Defense Action Set	Participant Action Set	Agent Action Set
Defense Strategy Set	Participant Strategy Set	Agent Strategy Set
Defense Decision Information Set	Participant Information Set	Agent Observation Information Set
Defense Decision Reward	Participant Utility	Agent Action Reward

**Table 4:** Experimental hyperparameter settings.

Modules	Parameters
PPO Algorithm	Number of layers for actor network: 3
	Number of layers for critic network: 3
	Number of nodes for actor layers: (128, 128, 64)
	Number of nodes for critic layers: (128, 128, 64)
	Learning rate: 2.5 $\times \;10^{-4}$
	The surrogate clipping coefficient: 0.2
	The number of mini-batches: 4
	The K epochs to update the policy: 4
Network Topology	Subnets: dmz_subnet, server_subnet, use_subnet
	dmz_subnet: 5 hosts
	server_subnet: 5 hosts
	use_subnet: 5 hosts
Attacker Action	Discovery: ping sweep, port scan
	Lateral Movement
	Privilege Escalation
	Impact
Defender Action	Deploy Decoy Host
	Remove Decoy
	Modify firewall_rules
	Patch Vulnerability
	nothing
Attacker Immediate & Recurring Reward	Discovery: (−2, 0)
	Lateral Movement: (−4, 0)
	Privilege Escalation: (−6, 0)
	Impact: (−8, −4)
Defender Immediate & Recurring Reward	Deploy Decoy Host: (−20, −2)
	Remove Decoy: (0, 0)
	Modify firewall_rules: (−10, 0)
	Patch Vulnerability: (−10, 0)
	nothing: (0, 0)

**Table 5:** Parameter settings of the Node2vec model.

Parameter	Description	Settings
Walk Length	Number of nodes traversed in a single random walk traversal	5 10 15 20
Embedding Dim	Dimension of the node characterization vector	32 64 128
In–out parameter *q*	Controls the probability of returning to a visited node	0.5 2
Return parameter *p*	Controls the balance between BFS and DFS traversal	0.5 2

## Data Availability

The data presented in this study are available on request from the corresponding author.

## Abbreviations

The following abbreviations are used in this manuscript:

ACD	Autonomous Cyber Defense
RL	Reinforcement Learning
PPO	Proximal Policy Optimization
BFS	Breadth-First Search
DFS	Depth-First Search
